# Authorized, clear and timely communication of risk to guide public perception and action: lessons of COVID-19 from China

**DOI:** 10.1186/s12889-021-11103-1

**Published:** 2021-08-12

**Authors:** Ni Gong, Xiaoyuan Jin, Jing Liao, Yundong Li, Meifen Zhang, Yu Cheng, Dong Xu

**Affiliations:** 1 School of Nursing, Jinan University, Guangzhou, P.R. China; 2 School of Public Health, Univeristy of Minnesota, Twin Cities, MN USA; 3 Department of Medical Statistics & Epidemiology, Sun Yat-sen Global Health Institute, School of Public Health, Sun Yat-sen University, No. 135 Xingang West Road, Guangzhou, P.R. China 510275; 4 Sun Yat-sen Global Health Institute, School of Public Health and Institute of State Governance, Sun Yat-sen University, Guangzhou, P.R. China; 5 Guangdong Academy of Social Sciences, Guangzhou, People’s Republic of China; 6 School of Nursing, Su, Yat-sen University, Guangzhou, P.R. China; 7 School of Sociology & Anthropology, Sun Yat-sen University, Guangzhou, P.R. China; 8 Global Health and Health Systems, School of Health Management, Southern Medical University, Guangzhou, China

**Keywords:** COVID-19, Risk communication, Information, Risk perception, Prevent measures, Lessons from China

## Abstract

**Backgrounds:**

This study examined the dynamic association between risk communication and the public’s risk perception and action across the COVID-19 outbreak timeline in China.

**Methods:**

This study collected publicly available information on COVID-19 released on official channels (e.g., government websites and official media) by the Parehub tool. Also, the study used the Zhongyun Big Data Platform to search public datasets about released COVID-19 information on Chinese social media, such as TikTok and Weibo. An online survey was conducted via WeChat to Chinese citizens using a snowball sampling method. The questionnaire assessed changes in participants’ risk perception and action against COVID-19. The data analysis examined information content and release-time trajectories against the public’s risk perception and actions over time.

**Results:**

Altogether, the collected data includes 1477 pieces of authorized information and 297,000 short videos on COVID-19. Of 1362 participants recruited from 33 provinces and municipalities of China, 1311 respondents (25–60 years, 42% male) were valid for future analysis. The study indicated that 85.7% of participants mainly relied on official channels to obtain information. Alongside the outbreak’s progress, there was a gradual rise in information quantity, publishing frequency, and content variation. Correspondingly, the public’s risk perception that “take it seriously” rose from 13 to 80%, 87.1% of those who took “multiple actions” compared to 25.9% initially.

**Conclusions:**

Our findings indicated that insufficient information freely-accessible at the early stages of the outbreak might lead to the lack of risk awareness and the public’s inadequate protective actions. Given the current global situation of COVID-19, the study highlights authorized, transparent, and timely two-way risk communication is vital to guide public perception and actions. Furthermore, our study provides risk communication recommendations and may contribute to developing full measures to address future crises.

## Background

On 31 December 2019, a new strain of coronavirus closely related to the one that causes Severe Acute Respiratory Syndrome (SARS) was discovered in Wuhan, Hubei, China [1, 2]. This new strain causes the 2019 coronavirus epidemic (COVID-19), and on 11 March 2020, WHO officially declared the disease as a global pandemic [3, 4]. Like SARS, the COVID-19 patients had clinical signs and symptoms like fever, cough, and difficulty breathing, and this new disease also spread through droplet transmission [4–6]. However, comparing with the SARS, COVID-19 is more urgent, with a higher fatality rate of 2.3% [7–9].

As a novel global pandemic, global researchers and scientists force exploring and investigating the treatments and medicine. However, the current public findings have not presented a specific vaccine that treats infectious ultimately, and professionals estimated the disease would maintain 18 months or longer [10, 11]. Additionally, the public has many worries about the COVID-19 vaccine; many people refused to take the vaccine, no matter its efficiency [9]. These studies analyzed the issue because the inequity between supply and demand about the vaccine, especially the business market on the demand side, is not computed [9]. Specifically, the government has not developed any COVID-19 vaccine profit programs to meet people with different socioeconomic levels. Therefore, countries preferred taking classic measures to prevent and control the pandemic and slow the spread compared with the vaccine promotion. Some studies also reviewed and analyzed sets of literature to state that suppression is the best possible method to slow and control the continuous spread of COVID-19 [10, 11]. Thus, many governments have imposed travel bans on an unprecedented scale to contain the transmission, close their borders, implement mandatory screening of citizens returning from heavily affected areas [12–17]. The lockdown policies also influence the education system; students, including international students, have to overcome the jet lag to take classes and receive an education using online technology platforms [10].

**Fig. 1:**
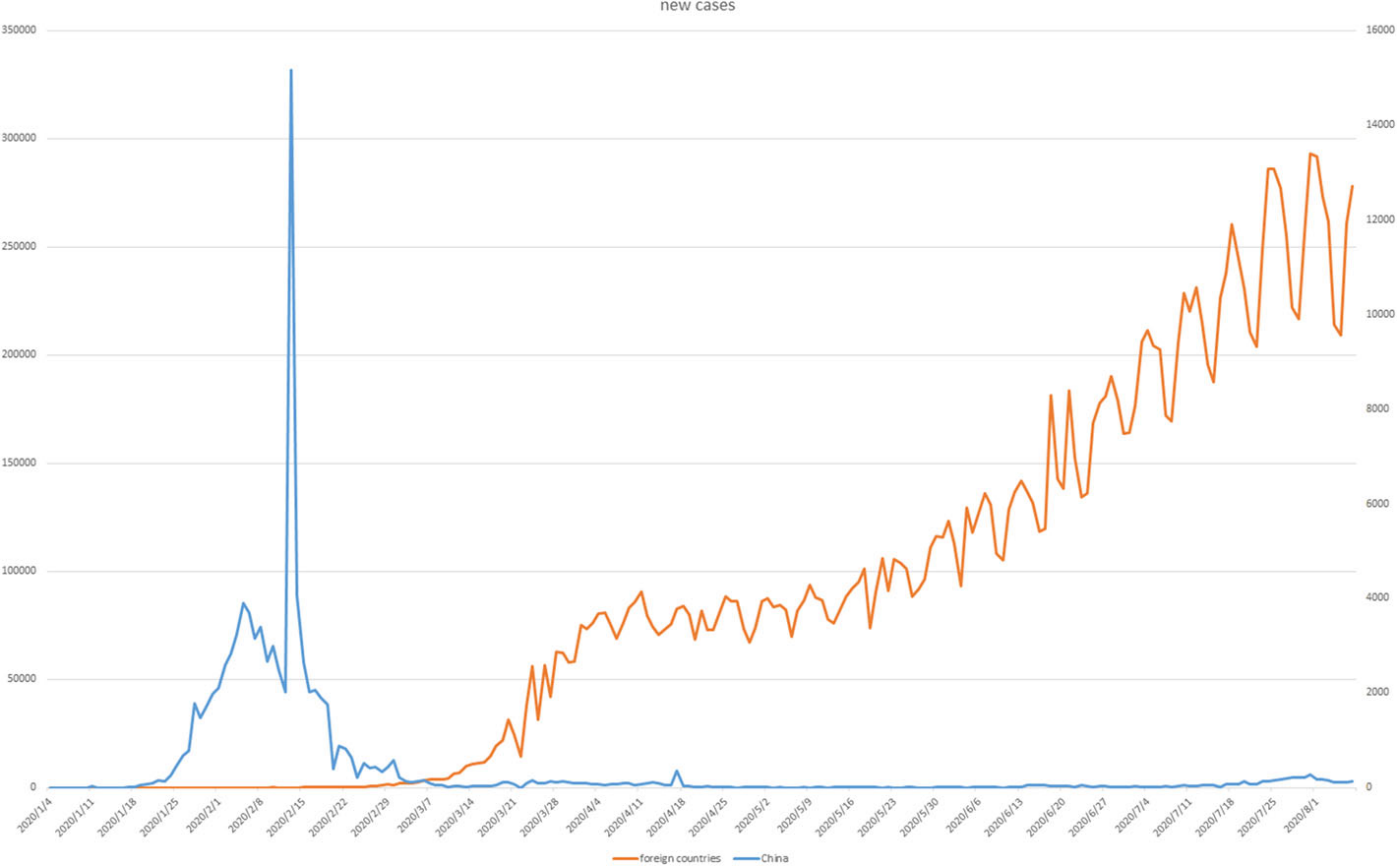
New cases reported weekly in China and other foreign countries

However, the current fact presents the pandemic is ongoing that hard to control. After 7 March 2020, the new incidence cases from many countries overgrow, and even the increased trend was hard to control that growth repeatedly when has the decreased cases (Shown in Fig. 1) [1, 18]. Notably, Fig. 1 also expressed, as the first country impacted by COVID-19, China has had steady declines in the number of new cases since March, and confirmed internal cases are controlled, even close to zero [1, 18]. The declined cases presented the Chinese government’s strict policy’s effectiveness, such as management by travel restrictions and home quarantine [14, 19]. Most importantly, as highlighted by the WHO, this achievement is impossible without the public’s collective willpower—to strict compliance with infection control policies [20].

The previous studies about SARS indicated that the individuals adopted more precautionary behaviors and followed preventive disease policies [19, 21, 22]. Based on this finding, this study considered the outbreak process in China as a lesson to explore the impacts from risk communications to risk perceptions among the public. The process examined the dynamic relationship between publicly- available information by its release channels, content and publish time, and the Chinese public’s risk perception and action across the outbreak timeline. The study aims to provide the global community with firsthand evidence of effective risk communication and guide public risk perception and self- protection.

## Methods

### Study design

#### Public data collection

Information on COVID-19 publicly- available was mainly released online through official channels (e.g., government websites and official media) and social media (e.g., TikTok, Weibo, etc.) in China [19]. We defined official channels as national and local health commissions websites, People’s Daily Online News, and CCTV news; and set the examination period from 8 December 2019 (the first reported pneumonia case) and 10 February 2020 (after the Chinese Lunar New Year). Using key terms “new coronavirus,” “epidemic,” and “pneumonia,” we extracted information related to disease (epidemic profile of the outbreak), preventive behaviors (preventative and measurements guidance), and social welfare (logistical and transportation support, etc.). By matching these words from JavaScript and AJAX pages, we collected data in seconds. Information released by official channels was compiled by the Parehub tool. Meanwhile, given recent studies on social media’s effects on risk communication about COVID-19 [23, 24], we searched public databases about released information in social media on Zhongyun Big Data Platform [25]. The collected data and dataset were all free-accessible, and thus did not require ethics approval.

#### Participant’s recruitment

Due to China’s national epidemic control strategies, it was not easy to reach and recruit participants in person. While the Chinese government also performed primary electronic services (telephone-based screening) to follow the citizens’ health status and identify the suspected patients [26]. For example, each person has a health code shown on the telephone, and the code’s colors changed with their location changes and moving trends. The different colors are designed and set to classify different health statuses and screen the suspected cases for medical observation in isolation wards. The support from the e-screening on the telephone provided an authorized check of our observed data resources’ daily health status and eliminated the recruited participants’ data bias [26]. Thus, the study conducted a web-based cross-sectional survey using the snowball sampling method from 4 February 2020 to 10 February 2020.

Undoubtedly, as the most critical communication channel in modern society, people rely on social media to receive information [27]. Also, considering the social media builds users’ social networks on sharing self-published posts and can read others’ sharing [27], the study applied the Wenjuanxing platform (https://www.wjx.cn/app/survey.aspx), a sub-function on the WeChat (the most popular social media in China), to spread the questionnaires. Specifically, researchers asked recruited participants to directly spread the links or “QR” codes about the electronic questionnaire to their WeChat contacts. Invalid surveys with incomplete information or respondents who were not staying in China during observed periods were excluded. Responses from a total of 1362 participants were received, 51 of which were invalid data, resulting in an adequate sample size of 1311 participants.

### Questionnaire design

**Fig. 2:**
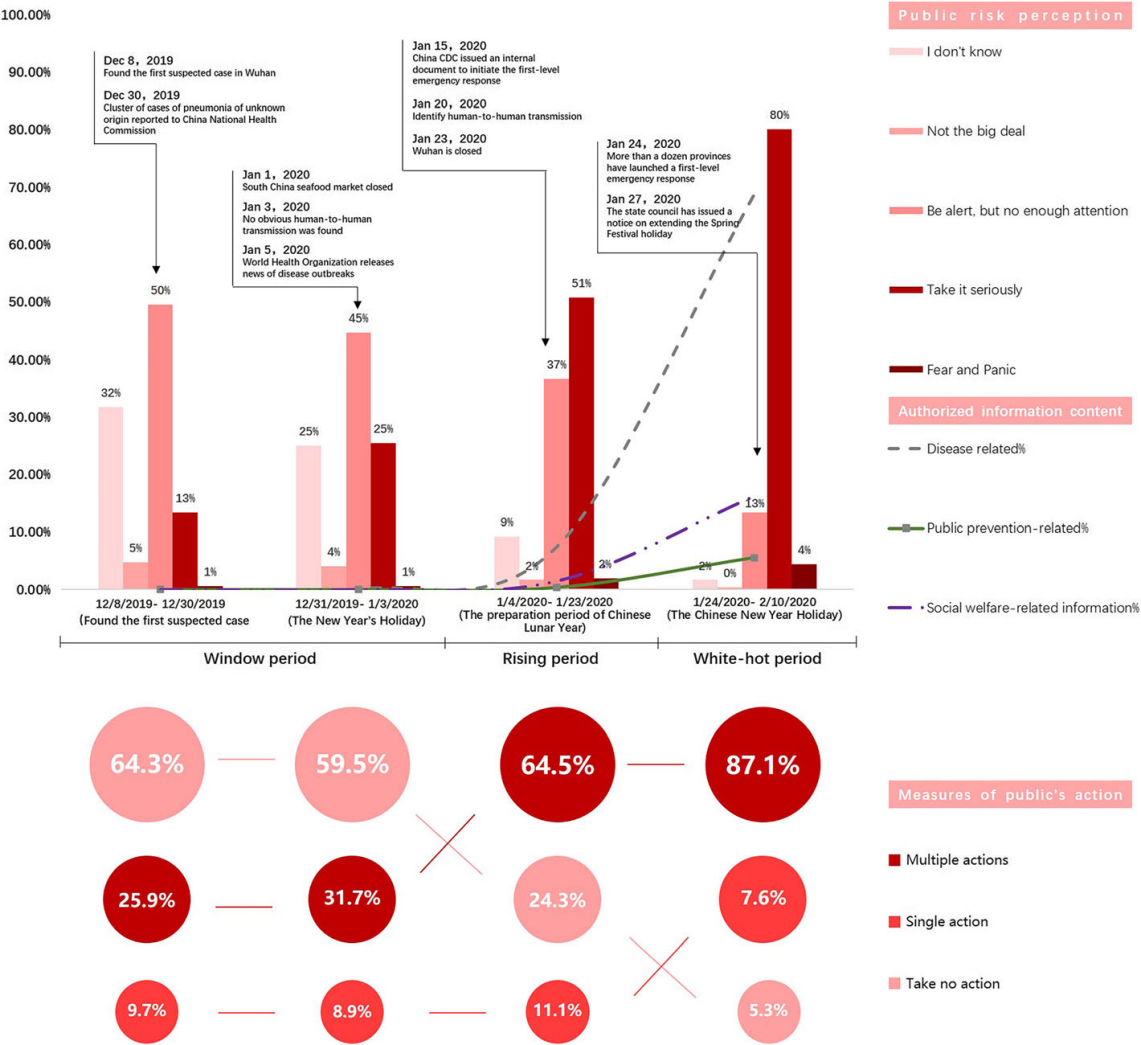
Type of authorized information provided, evaluated repeat measures of public risk perceptions and actions towards COVID-19 over time (8 Dec 2019–10 Feb 2020)

Because of the sudden outbreak of COVID-19, the study designed the questionnaire by reviewing much relevant literature related to SARS [19, 21]. Further, the questionnaire is finalized by consulting relevant experts for continuous improvement and modification. The questionnaire was designed into three parts specifically. The preliminary survey part collected all respondents’ demographic information, includes gender, marital status, age, education level, and monthly income. The second part of the survey measured participants’ trustworthiness of obtained information resources by asking questions about participants’ evaluations related to official channels and social media. The left survey questions repeatedly assessed participants’ trust in different information sources, risk perceptions, and prevention measures in four specific periods denoted by significant events of the outbreak or two National Holidays (Fig. 2, line chart).

### Ethics statement

The protocol was approved by the Institutional Review Board (IRB) of the Anthropology Department at Sun Yat-sen University (approve number: SYSUAD202003221). All of the participants have read and signed the electronic Informed Context before starting the survey.

### Statistical analysis

We calculated the number of publications to estimate the changes in trends. We also evaluated the percentage of the public’s perceived risk and measures taken versus their answered choices. Specifically, we observed their changes in trends by comparing each parameter’s percentage differences under different periods. Finally, we reached the changes in trends to that of published information, level of perceived risk, and measures to observe the effects among them.

## Results

### Data descriptions and participants characteristics

Altogether, the study retrieved 1477 pieces of authorized information on COVID-19 from the national and local health commissions websites (*n* = 180, 12%), People’s Daily Online News (*n* = 806, 55%), and CCTV news (*n* = 491, 33%) via the Parsehub tool. Over the same period, the collected dataset on Zhongyun Big Data included 297,000 pieces of short videos with the keyword “new coronavirus” and “suspected pneumonia,” which were released by mainstream Chinese social media and had been played over 21.8 billion times [18]. These channels were also selected as primary information sources by all respondents.

In total, 1311 respondents enrolled in this study were between the age of 25–60 years. Both sexes participated in the current research, including 57.7% females (*n* = 757). Besides, over half of the participants were married or cohabitated (*n* = 827, 63.1%). In terms of education level, 18.1% of the participants (*n* = 237) had a junior college education, 50% (*n* = 655) had a bachelor’s degree, and 17.9% (*n* = 234) had a master’s degree or above. As for monthly income, around half of the participants (*n* = 636, 48.5%) have steady income higher than 8000 Yuan each month (as shown in Table 1).

**Table 1:** Recruited participants’ demographical information

	Patients (*n* = 1311)
**Sex**	
Male	554 (42.3%)
Female	757 (57.7%)
**Age**	
≤ 25	258 (19.7%)
26 ~ 35	437 (33.3%)
36 ~ 45	282 (21.5%)
46 ~ 60	307 (23.4%)
> 60	27 (2.0%)
**Degree**	
High school/secondary school and below	185 (14.1%)
Junior college	237 (18.1%)
Bachelor	655 (50.0%)
Master’s degree and above	234 (17.9%)
**Income (yuan)**	
≥ 4000	199 (15.2%)
4001–8000	476 (36.3%)
< 8000	636 (48.5%)
**Family size (number of family member)**	
≤ 2	114 (8.7%)
3 ~ 5	1033 (78.8%)
> 5	164 (12.5%)
**Marriage status**	
Unmarried	443 (33.8%)
Married	827 (63.1%)
Divorced	35 (2.7%)
Widowed	6 (0.5%)

#### Trustworthiness of obtained information resources

The survey results showed that only 24% of responses chose to “obtain epidemic information” through TikTok, and only 4.8% believed that transmitted high credibility information. On the other hand, 85.76% of people chose to “obtain epidemic information” through “official news,” and 58.8% of participants tended to obtain the disease data through the “National Health Commission, Centers for Disease Control, and the official websites of hospitals.” 88.9% of the masses believed “official news” as a channel for obtaining highly credible information. Despite the high trust in authorized reports released by official channels, the contrasting amount of information released by official media versus social media heightens the possibility of information overload to the public.

#### Relationship between authorized information and public’s perceived beliefs

We further analyzed the content and released time of authorized information by official channels against the public’s perceived risk and actions. During the window period, as a new disease, the public’s knowledge about COVID-19 is low. These had less information publicly available to announce and promote the disease, so only two disease-related announcements were released by the Wuhan Municipal Health Commission. The rising period was during Chunyun, the World’s largest annual human migration. A gradual rise in COVID-19 disease-related information was most evident during this period, when the suspected and confirmed cases of COVID-19 had already accumulated to 830 and 1072, respectively, scattered over 29 cities across China. Information exploded over the white-hot period: 1338 pieces of data were released over 18 days, with 57 pieces of disease-related, 5 bits of public prevention-related, and 13 pieces of social welfare-related information were released per day.

The public’s risk perception towards COVID-19 was dominated by “I do not know” (45%) and “be alert, but not enough attention” (73%) during the window period (Fig. 1, bar chart). Even at the rising period, 37% of respondents still responded, “be alert, but not enough attention,” and only half of them started to “take it seriously.” Not until the white-hot period did the public start to pay attention to the epidemic. The public’s responses of “take it seriously” and “fear and panic” rose to 80 and 4%, while “be alert, but not enough attention” dropped to 13%, yet still ranked as the top two most prevalent responses. A similar lag in the public’s protective actions was also apparent (Fig. 2, bubble chart). Associated with the public’s low-risk perceptions, over two- thirds of the respondents chose to “take no actions” during the window period, which was about 2–2.5 times higher than those starting to take “multiple actions” (25%) (i.e., personal protection, self-isolation, and family education). This ratio reversed in the rising period, where 65% of respondents took “multiple actions.” and “take no actions” responses dropped to 24%. By the white-hot period, respondents predominately took “multiple actions” and increased by 61% compared to the initial period.

## Discussion

As the first country impacted by COVID-19, China’s success in containing this pandemic relies on the public’s high trust in, support of, and cooperation with the government [28]. Specifically, the Chinese government worked the functional crisis management with a lockdown policy to tell the world: the suspicion measurement is efficient for controlling disease spread [10, 11]. Further, the success of controlling new incidence cases includes the collective willpower from the public building under the success risk communications. The analysis of results indicated and provided three evidence-based recommendations for risk communication.

Initially, the public’s trust in health professionals, government agencies, and social media information sources can influence disease prevention’s perceived utility. As discussed in the previous literature about SARS, the public’s worry about epidemiological news and rumors [21]. Similarly, relating to the information about COVID-19, the study found that compared with social media (e.g., TikTok, WeChat), the participants prefer obtaining information from authorized channels, such as government official websites. This finding state that people have higher trust in expert, knowledgeable, and unbiased sources. Therefore, the study suggests the governments can explore diversity channels to hold risk communications with the public. For example, the public officials can prepare communities, risk managers, government spokespersons, hospital personnel to respond to crisis challenges.

Furthermore, in a risk communication situation, developing and updating timely information can improve the public’s perceived risk effectiveness. In reviewing the COVID-19 outbreak from the public’s perspective, it is seen that insufficient information publicly available at the early stages of the epidemic made it more difficult to confine the virus with the least amount of costs. However, when faced with the new disease, China provided specific disease data to the WHO and the whole world for seeking more information and findings of COVID-19. As the WHO mentioned, although the initial stage is the window period in China, the publication of data and information contributes to the world, showing that the goal is to earn more time and more experiences on preventing the disease [29]. On the other hand, the presented information related to brief and precise disease data helps improve the public’s perceived risk. Some studies found the people relied on receiving information from media, and they often judge personal risk based on their impressions of overall disease prevalence and severity [11, 30]. Studies have also shown that media reports about outbreaks that specify numbers of cases, hospitalizations, or deaths can influence avoidance behavior and contact patterns at both individual and community levels [11, 21, 30]. Thus, our study highlights that timely release of information related to the epidemic, specific prevention instructions, and updates are imperative to prevent the epidemic’s escalation.

Overall, our findings suggest the government should report the epidemic information logically and coherently, allowing the public, especially the elderly and other vulnerable populations, the time to be aware and to take appropriate protective actions as recommended. As firmly announced by the National Health Commission of the People’s Republic of China and the WHO: being first, being right, and being credible [28, 31].

The questionnaire results also noted that social media ever-increasingly becomes a critical channel to disseminate and reinforce information to the public, which should adequately utilize to provide accurate, non-contradictory, and easy-to-understand messages. Multiple information channels combined with modern technology may facilitate delivering a visualized and timely message [28].

This study has developed work in an iterative process. It referenced the body of work that addresses public risk communications’ effects on the individual’s perceived risk and preventive actions. It is acknowledged that our survey sample recruited online may mainly present participants prone to internet information, as most Internet-based questionnaire studies [30, 32]. While the internet is ever-becoming the primary channel for rapid and timely dissemination and access to information [33, 34], our study provides vital evidence to understand people’s trust in online information, thus better guiding them about preventing behaviors via mass media. For future studies, the authors recommend more rounds of cognitive interviews or focus groups to ensure the survey items are accessible and understandable to a wide variety of individuals. Additionally, follow-up interviews would flush out additional issues in the survey’s wording and structure. For the next step, it is necessary to confirm these relationships in the conceptual model by testing the survey.

## Conclusions

When facing the pandemic, countries worldwide need to formulate prevention and control strategies tailored to their situation, culture, and traditions. Those with sporadic cases should establish and maintain trust with the public via ongoing two-way communication and provide guidance to encourage the adaptation of protective behaviors. Simultaneously, while countries with clusters or community transmission should empowerresiliencein public by ongoing risk communications and nimble support addressing people’s concerns and needs. However, our study identified the cornerstone of these responses is the same for all countries, which is to engage the public’s responsive actions. Therefore, weathering the ongoing pandemic of COVID-19, our study urges countries worldwide to communicate the level of risk and prevention strategies to the public authoritatively, clearly, and timely.

Every disease outbreak is an opportunity for us to learn about giving a timely warning, optimizing public health advice, and providing frank communications. Numerous studies have highlighted lessons learned during and after disease outbreaks. However, how to send timely warnings, optimize public health advice, and provide frank communication opportunities remain at the forefront of what we must think about as we confront pandemics. Although the next pandemic is still unknown, regional epidemics have emerged with the intensification of globalization and population mobility, and strengthening risk communication has become an issue that cannot be ignored. Therefore, our research results may contribute to developing risk communication recommendations and full measures to address future crises.

## Data Availability

The data of survey results were conducted via the Wenjuanxing platform (https://www.wjx.cn/app/survey.aspx). More detailed data on participants’ reactions in different periods cannot be provided because of confidentiality policies of Wenjuanxing, and Anthropology Department at Sun Yat-sen University.

## Abbreviations

WHO: World health organization
COVID-19: The 2019 coronavirus epidemic
CCTV: China central television
